# Differential expression of glucose-metabolizing enzymes in multiple sclerosis lesions

**DOI:** 10.1186/s40478-015-0261-8

**Published:** 2015-12-04

**Authors:** Philip G. Nijland, Remco J. Molenaar, Susanne M. A. van der Pol, Paul van der Valk, Cornelis J. F. van Noorden, Helga E. de Vries, Jack van Horssen

**Affiliations:** Department of Molecular Cell Biology and Immunology, Neuroscience Campus Amsterdam, VU University Medical Center, P.O. Box 7057, 1007 MB Amsterdam, The Netherlands; Department of Cell Biology & Histology, Academic Medical Center, Meibergdreef 15, 1105 AZ Amsterdam, The Netherlands; Department of Pathology, VU University Medical Center, 1007MB Amsterdam, The Netherlands

**Keywords:** Glycolysis, TCA cycle, lactate shuttle, αKGDH, Neurodegeneration

## Abstract

**Introduction:**

Demyelinated axons in multiple sclerosis (MS) lesions have an increased energy demand in order to maintain conduction. However, oxidative stress-induced mitochondrial dysfunction likely alters glucose metabolism and consequently impairs neuronal function in MS. Imaging and pathological studies indicate that glucose metabolism is altered in MS, although the underlying mechanisms and its role in neurodegeneration remain elusive. We investigated expression patterns of key enzymes involved in glycolysis, tricarboxylic acid (TCA) cycle and lactate metabolism in well-characterized MS tissue to establish which regulators of glucose metabolism are involved in MS and to identify underlying mechanisms.

**Results:**

Expression levels of glycolytic enzymes were increased in active and inactive MS lesions, whereas expression levels of enzymes involved in the TCA cycle were upregulated in active MS lesions, but not in inactive MS lesions. We observed reduced expression and production capacity of mitochondrial α-ketoglutarate dehydrogenase (αKGDH) in demyelinated axons, which correlated with signs of axonal dysfunction. In inactive lesions, increased expression of lactate-producing enzymes was observed in astrocytes, whereas lactate-catabolising enzymes were mainly detected in axons. Our results demonstrate that the expression of various enzymes involved in glucose metabolism is increased in both astrocytes and axons in active MS lesions. In inactive MS lesions, we provide evidence that astrocytes undergo a glycolytic shift resulting in enhanced astrocyte-axon lactate shuttling, which may be pivotal for the survival of demyelinated axons.

**Conclusion:**

In conclusion, we show that key enzymes involved in energy metabolism are differentially expressed in active and inactive MS lesions. Our findings imply that, in addition to reduced oxidative phosphorylation activity, other bioenergetic pathways are affected as well, which may contribute to ongoing axonal degeneration in MS.

**Electronic supplementary material:**

The online version of this article (doi:10.1186/s40478-015-0261-8) contains supplementary material, which is available to authorized users.

## Introduction

Multiple sclerosis (MS) is an immune-mediated disease of the central nervous system where macrophages and T-cells infiltrate the brain and induce widespread demyelination [15]. Over time, the number of newly-formed lesions decreases and disease progression seems to be mainly driven by axonal dysfunction and neuronal degeneration [13]. Evidence is emerging that mitochondrial dysfunction and associated oxidative stress play an important role in driving neurodegeneration [25, 47]. Demyelinated axons have to consume more energy to maintain conduction [42]. As a result, axons in MS lesions contain more mitochondria, but, strikingly, lower oxidative phosphorylation (OxPhos) activity and increased numbers of mtDNA deletions in the neuronal cell bodies, indicating that mitochondria are damaged and dysfunctional [8, 46]. The OxPhos system is the last step in glucose metabolism and is critically dependent on glycolysis and the tricarboxylic acid (TCA) cycle to provide the electrons needed to maintain a proton gradient and to produce ATP. MS patients have elevated levels of essential glucose metabolites in the cerebrospinal fluid (CSF), serum and the brain as compared to non-neurological controls [39, 41, 50]. Although it remains difficult to determine which cells are responsible for these changes in the patient’s fluids, these studies illustrates that bioenergetic changes likely occur in MS patients. Moreover, glucose and lactate levels are increased in MS lesions, as determined with positron emission tomography (PET) and magnetic resonance spectroscopy (MRS) [39, 40]. Taken together, there is ample evidence that glucose metabolism is altered in MS brain tissue and may play an important role in driving neurodegeneration.

To date, most studies have focussed on OxPhos activity and several studies demonstrated profound defects in OxPhos activity in MS. Yet, surprisingly little is known about the expression of enzymes involved in glycolyis and TCA cycle flux in MS tissue. Glycolysis is the metabolic pathway in which glucose is metabolised into pyruvate by various enzymes, including the rate-limiting enzymes hexokinase (HK) and pyruvate kinase (PK) [2]. There are three different isoforms of HK of which hexokinase 2 (HK2) has been shown to be the principal regulated isoform [48]. HK2 rapidly phosphorylates glucose into glucose-6-phosphate, which is the initial step of the glycolysis, while the final step of the glycolysis is catalysed by PK [48, 49]. Pyruvate produced by glycolysis can be transported into mitochondria where it is converted into acetyl-CoA by pyruvate dehydrogenase (PDH) to fuel the TCA cycle. Two other rate - limiting TCA cycle enzymes are α-ketoglutarate dehydrogenase (αKGDH) and malate dehydrogenase (MDH), both producing NADH [17, 19, 33]. NADH is the most important driving force of OxPhos, intimately linking TCA cycle metabolism and OxPhos.

In addition to glucose, lactate can serve as an important energy source in the brain. Lactate can be produced from pyruvate by lactate dehydrogenase (LDH), which forms 5 different multiprotein complexes consisting of the products of two genes; *LDHA* and *LDHB*. The different complexes vary in the abundance of LDHA and LDHB. LDHA is mainly involved in the conversion of pyruvate into lactate, whereas LDHB preferably generates pyruvate from lactate [26].

It has been proposed that lactate secreted by astrocytes via specific monocarboxylate transporters (MCT) can be taken up and oxidized into pyruvate by neighbouring cells [36]. This metabolic coupling, known as the astrocyte-neuron lactate shuttle (ANLS), seems to be of particular importance in grey matter [34]. In white matter, oligodendrocytes rather than astrocytes supply axons with lactate via MCT1, which is essential for proper axonal function [16, 22].

Brain glucose metabolism is subjected to changes during aging and an altered glucose metabolism has been suggested to contribute to neurodegenerative disorders including Alzheimer’s disease (AD) and Huntington disease (HD) [11, 20]. It has been shown that the activity and expression of various metabolic enzymes was altered in the brain of AD patients [5, 7, 37]. Experimental studies have demonstrated that increased glycolytic activity induces a pro-inflammatory phenotype in astrocytes and can induce cell death in neuronal cells [38, 44]. Moreover, reduced activity of specific TCA cycle enzymes such as PDH and αKGDH, have been associated with neurodegeneration [32, 43]. These studies illustrate the impact of altered glycolysis and TCA cycle function on brain functionality and neurodegeneration.

To unravel the mechanisms underlying neurodegeneration in MS, we aimed to gain more insight into glucose metabolism in MS lesions. We show that expression of glycolytic and TCA cycle enzymes is highly increased in active MS lesions. In inactive MS lesions, astrocytes evidently increase the expression of key glycolytic and lactate-producing enzymes, whereas axons mainly upregulate lactate-catabolising enzymes. Finally, we observed reduced expression and reduced NADH production capacity of mitochondrial αKGDH in demyelinated axons, which correlates with axonal dysfunction. Taken together, we provide evidence for increased glycolysis, increased astrocyte-axon lactate coupling and decreased axonal mitochondrial function in MS, which may contribute to the ongoing axonal degeneration.

## Materials and methods

### Brain tissue

Brain paraffin tissue samples of 14 MS patients and 4 non-neurological controls were obtained in collaboration with the Netherlands Brain Bank, Amsterdam, The Netherlands (coordinator Dr. I. Huitinga). In addition, frozen tissue blocks were obtained from 6 MS patients and 3 non-neurological controls for histochemical analysis. Detailed clinical data are summarized in Table 1. The study was approved by the institutional ethics review board (VU University Medical Center, Amsterdam, The Netherlands) and all donors or their next of kin provided written informed consent for brain autopsy, use of material and clinical information for research purposes. Lesion types were determined by Proteolipid protein (PLP) and MHCII staining and are summarized in Table 1.

**Table 1:** Clinical data of MS patients and non-neurological controls

Case	Age (years)	Type of MS	Sex	Post-mortem delay (h:min)	Disease duration (years)	Lesion stages	Tissue
1	51	SP	m	11:00	>15	A	P/F
2	56	SP	m	8:00	27	CA	P
3	49	RR-SP	m	8:00	25	CA	P
4	66	SP	f	6:00	23	CA	P/F
5	54	ND	m	8:30	ND	CA	P
6	66	PP	m	7:30	26	CA	P
7	41	PP	m	7:23	14	A	P
8	54	ND	m	8:30	15	A	P
9	75	RR-SP	m	7:45	24	CIA	P
10	70	SP	f	6:55	40	CIA	P
11	44	PP	m	12:00	16	CA, A	P
12	50	RR-SP	f	7:35	17	CA	P
13	61	SP	m	9:15	14	CA	P
14	64	RR-SP	f	10:10	38	CIA	P
15	70	RR-SP	m	7:45	46	CIA	F
16	77	PP	m	4:15	26	A	F
17	48	SP	f	11:40	23	CIA	F
18	56	SP	m	10:10	13	CA	F
19	84	control	f	6:55	NA	NA	P
20	56	control	m	9:15	NA	NA	P
21	62	control	m	7:20	NA	NA	P
22	82	control	m	6:20	NA	NA	P
23	55	control	m	7:30	NA	NA	F
24	76	control	m	6:45	NA	NA	F
25	70	control	f	6:15	NA	NA	F

*SP* secondary progressive MS, *PP* primary progressive MS, *ND* not determined, *NA* not applicable, *m* male, *f* female, *A* active lesion, *CA* chronic active lesion, *CIA* chronic inactive lesion, *P* paraffinembedded tissue, *F* frozen tissue

### Immunohistochemistry

Immunohistochemistry was performed as described previously [29]. In short, 5 μm-thick paraffin sections were deparaffinised in a series of xylene and ethanol. Endogenous peroxidase activity was blocked by incubating the sections in methanol containing 0.3 % H_2_O_2_ for 30′ and the antigens were retrieved in citrate buffer (pH 6). Primary antibodies (see Additional file 1: Table S1) were diluted in phosphate-buffered saline (PBS) supplemented with 1 % bovine serum albumin (BSA; Roche Diagnostics, Mannheim, Germany) and 0.05 % Tween-20 (SigmaAldrich, St. Louis, MO) and the sections were incubated overnight at 4 °C. The next day, sections were incubated for 30′ with EnVision secondary antibody coupled with horseradish peroxidase (DAKO, Glostrup, Denmark) followed by 10′ incubation in the presence of 3,3′diaminobenzidine-tetrahydrochloridedihydrate (DAB; DAKO). Sections were washed in PBS for at least 3 × 5′ in between steps. Sections were counterstained with haematoxylin for 1’, rinsed with tap water and dehydrated in a series of ethanol and xylene and mounted with Entellan (Merck, Darmstadt, Germany). Negative controls were essentially blank. DAB stainings were semi-quantitatively, independently and blind analyzed by PN and JvH.

Fluorescence immunohistochemistry was applied to indentify cellular localization patterns. For this purpose, deparaffinised sections were incubated for 20′ in PBS containing 1 % BSA, 0.05 % Tween-20 and 10 % normal goat serum followed by incubation with primary antibodies. Alexa Fluor® (Life Technologies, Vienna, Austria) labelled secondary antibodies were used for fluorescence labelling. To reduce autofluorescence, sections were counterstained with Sudan Black (0.3 % in 70 % ethanol) (SigmaAldrich). Finally, sections were stained with Hoechst (diluted 1:1.000) (Life Technologies) to visualize cellular nuclei and mounted with mounting medium (DAKO). Images were taken using a confocal microscope (TCS SP2, Leica Microsystems, Mannheim, Germany) equipped with an Ar/Kr laser (488 nm) and 3 HeNe lasers (543, 594 and 633 nm).

In order to quantify axonal colocalization, 9 images of the active rim and 9 images of the inactive lesion center were obtained, each of 3 different MS patients. All images were taken with a 40x objective except the images of αKGDH expression in axonal mitochondria which were taken with a 63x objective. The images were analyzed with ImageJ software, using the intensity correlation plug-in [23].

### Quantitative enzyme histochemistry

Frozen sections (5 μm) were stained using metabolic mapping to visualize NAD^+^-dependent activity of lactate dehydrogenase (LDH; EC number 1.1.1.27) and αKG dehydrogenase (αKGDH; EC number 1.2.4.2). Quantitative enzyme activity experiments were conducted and analyzed as described previously [9, 28]. Incubation in the presence of appropriate substrate and cofactors was performed at 37 °C for 60'. Control reactions were performed in the absence of substrate, but in the presence of cofactors to control for non-specific enzyme activity staining [9, 28].

Monochromatic images were taken from the active rim (*N* = 12) and inactive lesion center (*N* = 15) of 5 different MS patients for quantification using the Nuance® spectral imager (PerkinElmer, Waltham, MA) at a wavelength of 585 nm. The total area positively stained was calculated using ImageJ software. Control reactions for LDH were essentially blank. αKGDH activity was relatively low resulting in higher background staining. Therefore, the total area measured in control sections was subtracted from the total area positively stained in the presence of the substrate.

### Cell culture and treatment

The human neuroblastoma cell line SH-SY5Y was cultured in DMEM/F12 (1:1, Life Technologies) containing 10 % fetal calf serum (FCS, Life Technologies), 2 mM L-glutamine (Life Technologies), and penicillin/streptomycin (50 mg/ml; Life Technologies) in 5 % CO_2_ at 37 ° C. Various treatment strategies were used to unravel neuronal αKGDH regulation. Cells were cultured for 24 h in glucose-free medium supplemented with 25 mM or 5 mM glucose to generate hypoglycaemic conditions. Alternatively, confluent SH-SY5Y cells were cultured in a hypoxic chamber in the presence of 2 % oxygen for 24 h. Finally, cells were exposed to 25 μM tert-butyl hydrogen peroxide (tbH_2_O_2_) (SigmaAldrich) or tumour necrosis factor α (TNF-α) and interferon γ (IFN-γ) (5 ng/ml; Peprotech, Rocky Hill, NJ) for 24 h.

### RNA isolation and quantitative PCR

Total RNA from SH-SY5Y cells was isolated using Trizol (Invitrogen, Carlsbad, CA) according to the manufacturer’s protocol. cDNA was synthesized with the high-capacity cDNA reverse transcription kit (Applied Biosystems, Foster City, CA) following manufacturer’s guidelines. Quantitative PCR (qPCR) reactions were performed in a step-one sequence detection system using the SYBR Green method (Applied Biosystems) as described previously [27]. mRNA expression levels were normalized to the household gene XPNPEP1 (Qiagen, Venlo, the Netherlands) expression levels [12]. αkGDH primers (forward: GGGATTTTGGATGCTGATCTG, reverse: AGTGGAAGACCTTGTCGAG) were synthesized by Invitrogen.

### Statistical analysis

One-way ANOVA with Bonferroni post-hoc test was used to assess differences in colocalization between the normal appearing white matter (NAWM), active rim and inactive lesion center. The student’s *t*-test was used to assess differences in the level of enzyme staining intensity in active and inactive MS lesions (Fig. 3j), αKGDH staining (Fig. 5I) and αKGDH activity (Fig. 5l). Linear regression analysis was used to determine the correlation between TCA cycle gene expression and disease duration.

## Results

### Increased expression of key glycolytic enzymes in active and inactive MS lesions

**Fig. 1:**
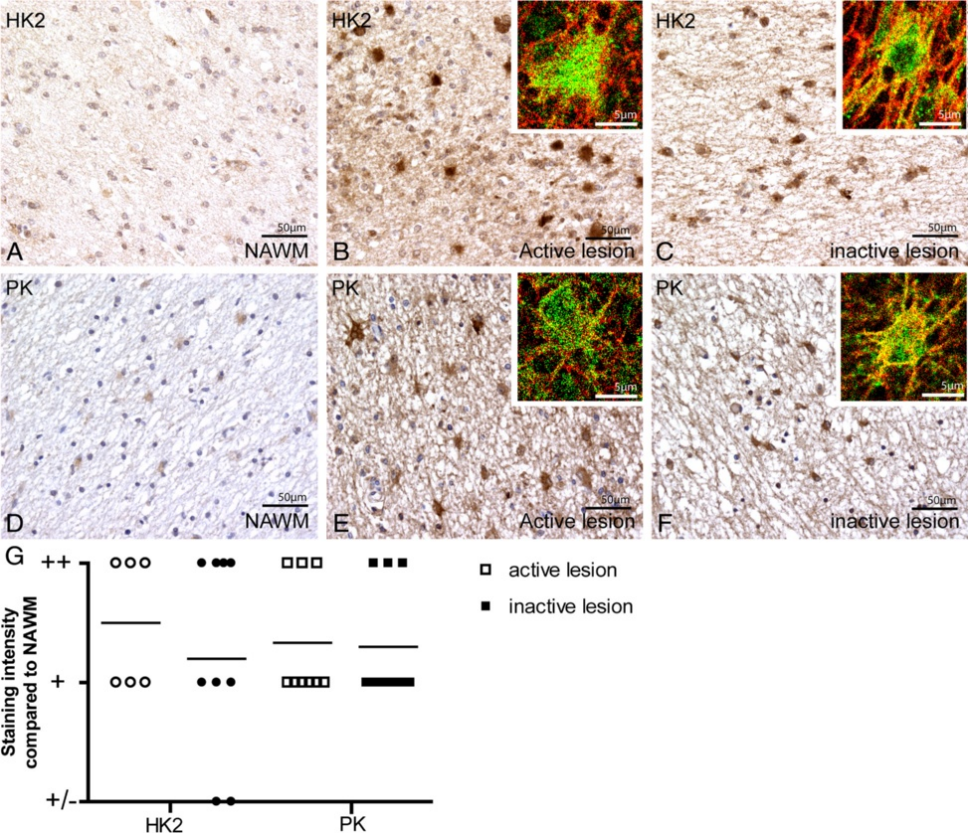
HK2 and PK expression in MS lesions. HK2 and PK are weakly expressed throughout the NAWM (**a**, **d**). Expression of both HK2 and PK was more pronounced in active and inactive MS lesions (**b**-**c**, **e**-**f**). Insets show immunofluorescence labelling of GFAP (red) and HK2 (green, **b**-**c**) or PK (green, **e**-**f**) indicating that astrocytes in MS lesions express high levels of HK2 and PK. Staining intensity of HK2 and PK2 in MS lesions was increased as compared to NAWM in the same section (**g**). +/− = similar staining intensity as compared to NAWM, + = increased staining intensity as compared to NAWM, ++ = strongly increased staining intensity as compared to NAWM

**Fig. 2:**
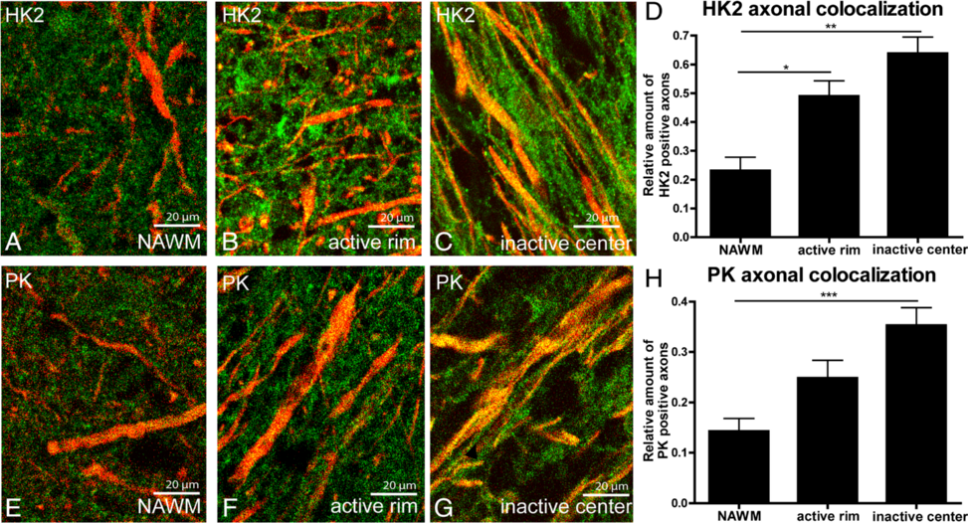
Axonal HK2 and PK expression in MS lesions. Immunofluorescent double staining of HK2 (**a**-**c**, green) or PK (**e**-**g**, green) and axons, labelled by the pan-neurofilament marker SMI312 (red), illustrates increased HK2 and PK axonal colocalization. The number of colocalizing pixels was quantified (**d**, **h**). *P < 0.05, **P < 0.01, ***P < 0.001

Active MS lesions are characterized by the presence of densely packed macrophages throughout the lesion area, whereas inflammation has abated in inactive MS lesions (Additional file 2: Figure S1). Expression of HK2 and PK was strikingly upregulated in both active and inactive MS lesions as compared to surrounding normal appearing white matter (NAWM), and predominantly localized in reactive astrocytes (Fig. 1a-f). Marked differences were not observed in HK2 and PK immunoreactivity between control white matter and NAWM (data not shown). HK2 and PK staining intensity in MS lesions as compared to NAWM was semi-quantitatively scored and revealed a striking increase in HK2 and PK immunoreactivity in both active and inactive MS lesions as compared to NAWM (Fig. 1g). Chronic active lesions are characterized by a rim of activated microglia and macrophages and a center devoid of inflammatory cells (Additional file 2: Figure S1). The expression pattern and staining intensity of HK2 and PK in the active rim of chronic active lesions was comparable to that observed in active lesions. Likewise, HK2 and PK expression levels in the inactive center of chronic active lesions were similar as observed in inactive lesions (data not shown). Sections containing chronic active lesions were used to quantify the cellular colocalization of metabolic enzymes allowing the comparison between active and inactive lesion within the same section. Immunofluorescence double labelling of neurofilament and HK2 and PK showed significantly increased HK2 and PK expression in demyelinated axons, most notably in the inactive center of chronic active MS lesions (Fig. 2).

### Increased astrocyte-axon lactate coupling in inactive MS lesions

**Fig. 3:**
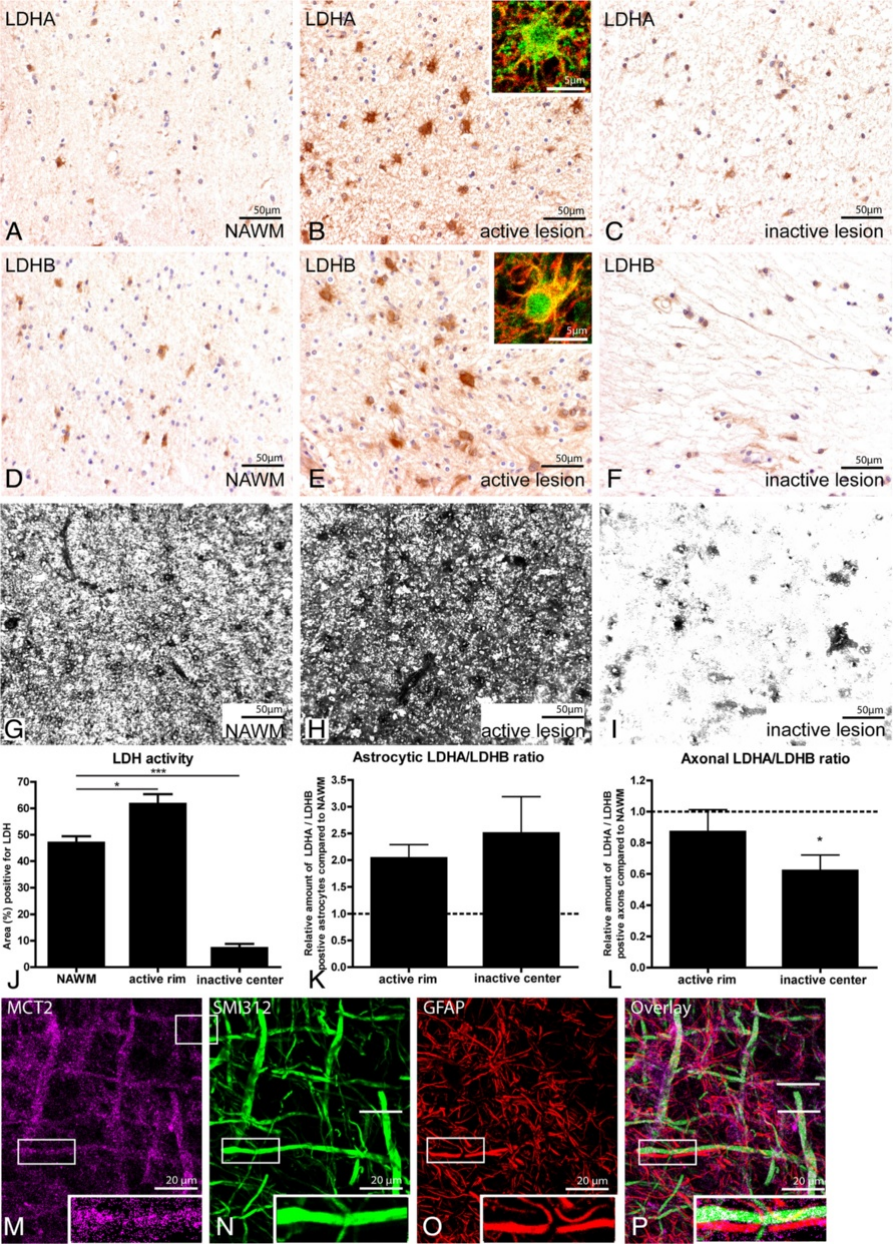
Astrocyte-axon lactate coupling in MS lesions. LDHA and LDHB proteins were moderately expressed in the NAWM (**a**,**d**) and inactive MS lesions (**c**,**f**). In active MS lesions intense LDHA (**b**) and LDHB (**e**) immunoreactivity was observed. Insets show imunnofluorescence labeling of LDHA (**b**, green) and LDHB (**e**) with the astrocytic marker GFAP (red). NAD^+^-dependent LDH activity was increased in active MS lesions but decreased in inactive lesions compared to NAWM (**g**-**i**). The quantitative data are shown in (**j**). Quantification of immunofluorescence double labeling of LDHA and LDHB with respectively GFAP (**k**) or SMI312 (**l**) in lesions as compared to NAWM. Staining of MCT2 (**m**, magenta), SMI312 (**n**, green), GFAP (**o**, red) and corresponding overlay (**p**) in inactive MS lesions. *P < 0.05, ***P < 0.001

Increased expression of HK2 and PK in inactive MS lesions is indicative of a high glycolytic rate, which is generally associated with increased production and secretion of lactate [45, 48]. Therefore, we aimed to determine lactate production in MS lesions. Lactate is produced by LDH, which forms a multiprotein complex consisting of LDHA and LDHB. LDHA is responsible for lactate production and LDHB for lactate utilization. LDHA and LDHB immunoreactivity was evidently increased in active MS lesions as compared to surrounding NAWM. Anti-LDHA and anti-LDHB antibodies predominantly decorated astrocytes (Fig. 3a-b,d-e). LDHA and LDHB expression levels were comparable in inactive lesions and NAWM (Fig. 3c,f). Using quantitative enzyme histochemistry, we found that the activity of lactate utilization by LDH was highly increased in active lesions, but decreased in inactive lesions (Fig. 3g-j). Next, we determined the ratio of LDHA/LDHB expression in MS lesions in order to determine whether lactate is rather produced or utilized in astrocytes and axons. The astrocytic LDHA/LDHB ratio was increased in inactive lesions compared to the NAWM (Fig. 3k). In contrast, the ratio of LDHA/LDHB immunoreactivity was decreased in axons in the inactive lesion center of MS lesions as compared to NAWM (Fig. 3l). Thus, in inactive MS lesions, astrocytes express more lactate-producing enzymes, whereas axons express more lactate-degrading enzymes.

Under normal conditions oligodendrocytes supply axons with lactate [16, 22]. Here, we hypothesized, that in the absence of oligodendrocytes, astrocytes provide demyelinated axons with lactate. We previously described that astrocytes in inactive MS lesions express increased levels of MCT1, which is involved in the secretion of lactate [29]. Axons require MCT2 to take up lactate. Here we found that MCT2 is expressed by demyelinated axons that are in close contact with astrocytes (Fig. 3m-p) [29].

In summary, our data demonstrates increased expression of glycolytic and lactate-producing enzymes in astrocytes in the inactive center of chronic active MS lesions. Demyelinated axons have increased expression of lactate-consuming enzymes and are able to take up lactate.

### Increased expression of key TCA cycle enzymes in active MS lesions

**Fig. 4:**
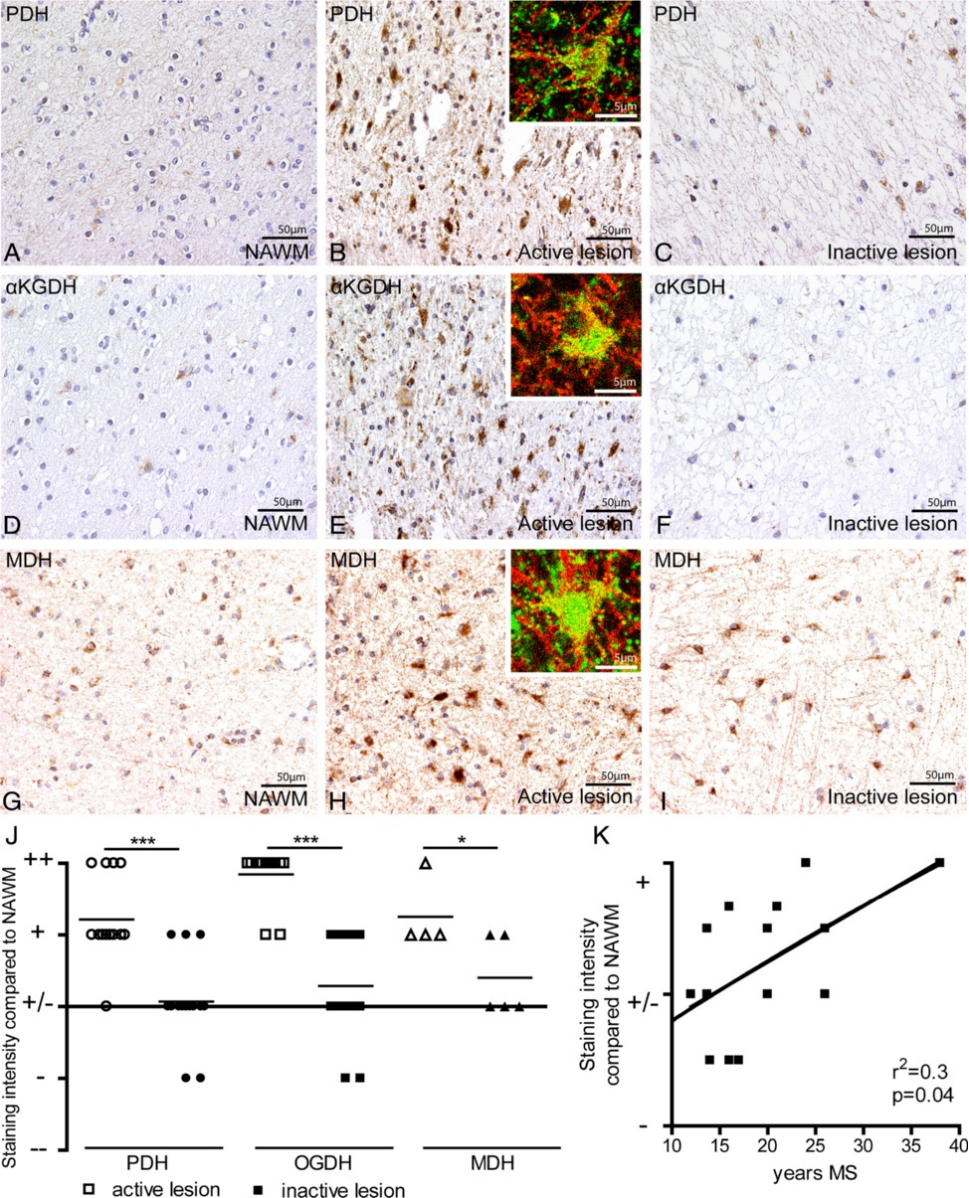
PDH, αKGDH and MDH expression in MS lesions. PDH (**a**, **b**), αKGDH (**d**,**e**) and MDH (**g**,**h**) expression was increased in active MS lesions as compared to NAWM. Insets show that GFAP-positive astrocytes (red) colocalize with respectively PDH (**b**), αKGDH (**e**) and MDH (**i**, green). PDH (**c**) and αKGDH (**f**) expression was relatively low in inactive MS lesions, whereas MDH (**i**) expression was increased as compared to NAWM. The staining intensity of PDH, αKGDH, and MDH was significantly reduced in inactive lesions and the inactive center of chronic active lesions as compared to active lesions and the active rim of chronic active lesions (**j**). PDH, αKGDH, and MDH immunoreactivity correlates with disease duration (**k**). -- = strongly reduced immunoreactivity as compared to NAWM; − = reduced immunoreactivity as compared to NAWM; +/− = similar immunoreactivity as compared to NAWM, + = increased immunoreactivity as compared to NAWM, ++ = strongly increased immunoreactivity as compared to NAWM. *P < 0.05, ***P < 0.001

Expression of TCA cycle enzymes PDH, αKGDH and MDH was consistently increased in active MS lesions as compared to surrounding NAWM and is localized predominantly in astrocytes (Fig. 4a-b, d-e, g-h). No marked differences were observed in PDH, αKGDH and MDH immunoreactivity between control white matter and NAWM (data not shown). Expression levels of PDH and αKGDH were similar in inactive MS lesions and NAWM (Fig. 4c,f). In contrast, MDH immunostaining was slightly increased in inactive lesions compared to NAWM (Fig. 4i). Semi-quantitative analysis demonstrated that expression of TCA cycle enzymes was increased specifically in active MS lesions (Fig. 4j). The staining intensity of PDH, αKGDH and MDH in inactive MS lesions correlated with disease duration (Fig. 4k), but was independent of the age of the patient or MS type (Additional file 3: Figure S2).

**Fig. 5:**
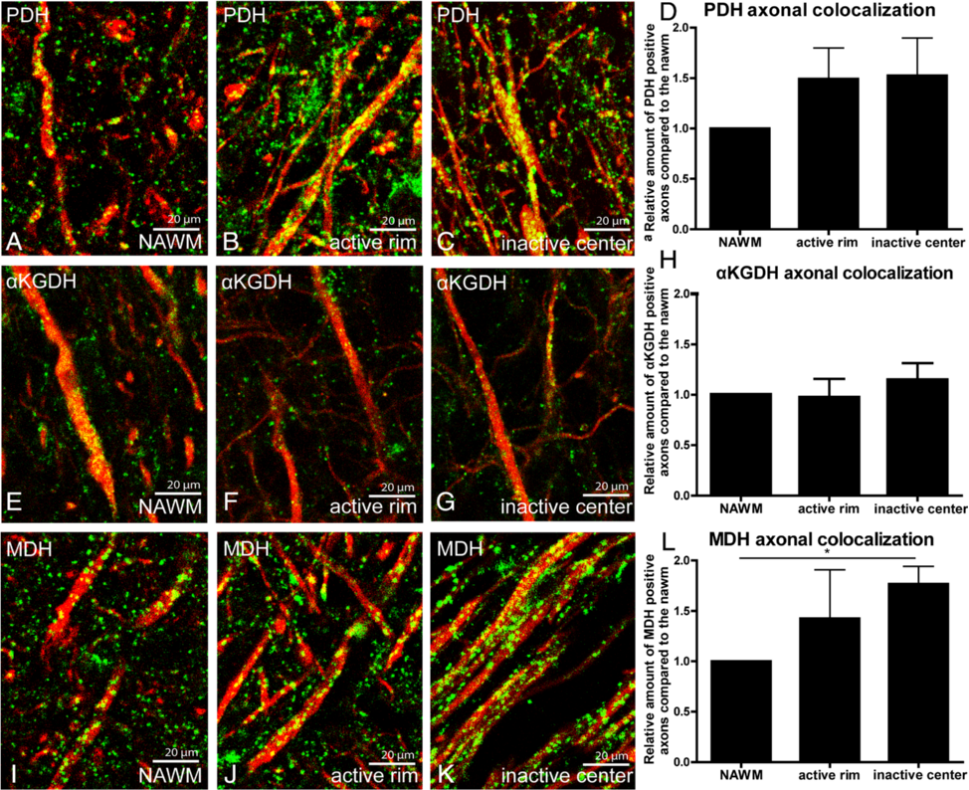
Axonal PDH, αKGDH and MDH expression in MS lesions. Immunofluorescence labelling of PDH (**a**-**c**), αKGDH (**e**-**g**) and MDH (**i**-**k**) in green and SMI312-positive axonal structures in red. The axonal colocalization of PDH (**d**), αKGDH (**h**) and MDH (**l**) was quantified. *P < 0.05, **P < 0.01

Axonal localization of both PDH and MDH was increased in the active rim and inactive lesion center of chronic active MS lesions as compared to NAWM (Fig. 5a-l). In contrast, differences in axonal αKGDH expression were not observed when comparing active and inactive lesion areas with NAWM (Fig. 5e-h).

Taken together, these results indicate that the expression of key glycolytic and TCA cycle enzymes is strongly increased in the active rim of MS lesions. In the inactive lesion center, expression of glycolytic enzymes is increased in both astrocytes and axons, whereas the TCA cycle enzymes show a differential expression pattern.

### Reduced axonal αKGDH expression and activity in MS lesions

**Fig. 6:**
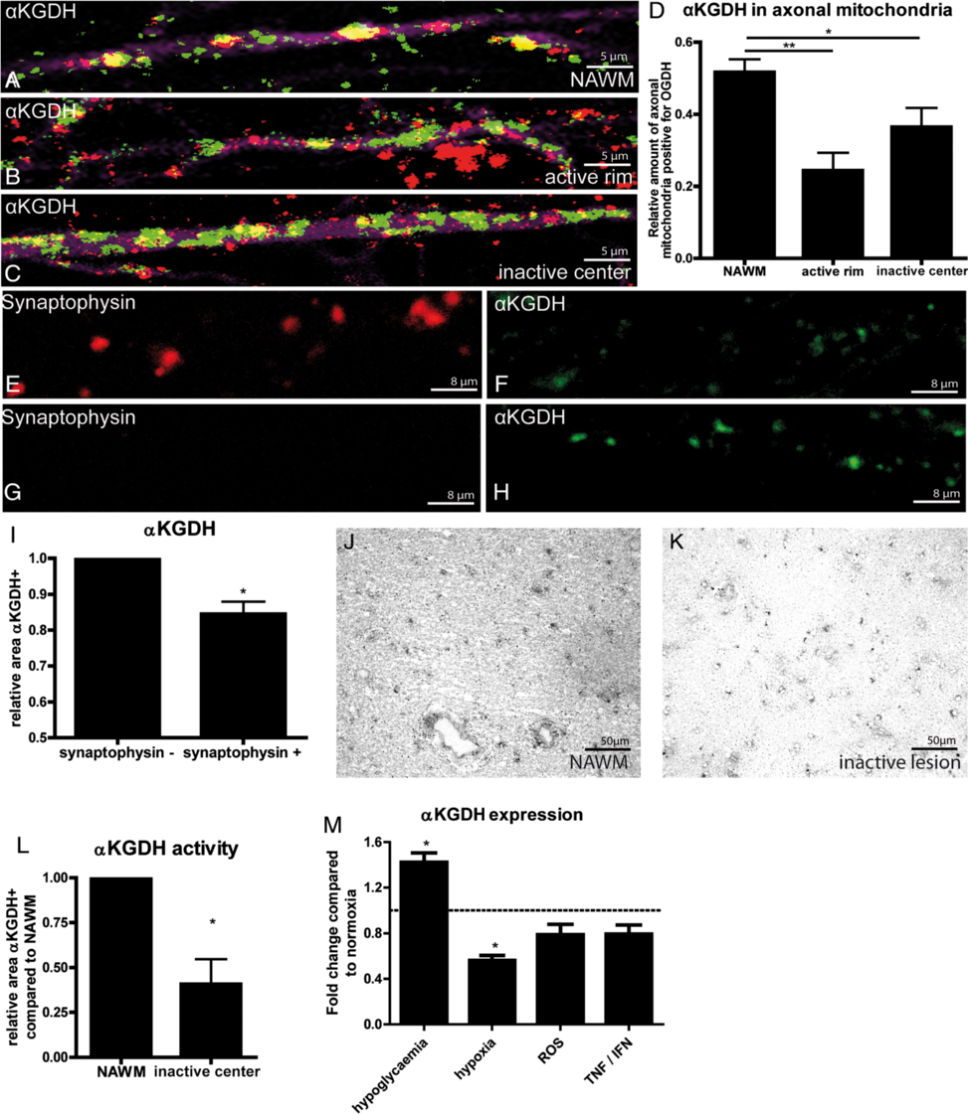
Axonal αKGDH expression, regulation and activity in MS lesions. High magnification immunofluorescence staining of αKGDH (red), porin (green) and SMI312 (magenta) (**a**-**c**). The quantitative data are shown in (**d**). Double labelling of synaptophysin (**e**,**g**, red) and αKGDH (**f**, **h**, green). The total area stained for αKGDH was determined in synaptophysin-positive and synaptophysin-negative images (**i**). NAD^+^-dependent αKGDH activity was reduced in inactive lesions (**k**) as compared to NAWM (**j**). The quantitative data are shown in (**l**). Culture of human neuroblastoma cells under hypoxia or in the presence of ROS or TNF-α and IFN-γ reduced αKGDH mRNA expression (**m**). *P < 0.05, **P < 0.01

In line with previous studies, we observed increased expression of the mitochondrial marker porin in astrocytes and axons in both active and inactive lesions (data not shown) [24, 46]. Despite an increased number of mitochondria, αKGDH expression remained unaltered, suggesting that αKGDH levels are reduced in axonal mitochondria. Triple immunofluorescence staining of αKGDH, porin and SMI312 demonstrated that αKGDH expression was significantly reduced in mitochondria in demyelinated axons as compared to mitochondria in myelinated axons in NAWM (Fig. 6a-d). Importantly, mitochondrial αKGDH expression was reduced in dysfunctional axons that were identified by synaptophysin accumulation (Fig. 6e-i) (Additional file 4: Figure S3). Quantitative enzyme histochemistry demonstrated that αKGDH NADH production capacity was markedly decreased in inactive lesions (Fig. 6j-l) (Additional file 4: Figure S3).

To determine which factors underlie reduced axonal expression and NADH production capacity of αKGDH in MS lesions, we cultured human neuroblastoma cells under various conditions. Demyelinated axons in MS lesions are considered to be in a state of virtual hypoxia [42]. Therefore, we cultured neuronal cells under hypoglycaemic and hypoxic conditions. Hypoglycaemia increased αKGDH mRNA levels, whereas hypoxia significantly reduced αKGDH expression (Fig. 6m). Treatment for 24 h with TNF-α and IFN-γ, pro-inflammatory cytokines that are abundantly present in active MS lesions, resulted in a small, but consistently decreased αKGDH mRNA expression [3, 6]. Reactive oxygen species are considered to play an important role in mitochondrial dysfunction and associated axonal degeneration [30]. We treated neurons with tertbutyl hydrogen peroxide (tbH_2_O_2_) to induce oxidative stress and found slightly decreased αKGDH levels (Fig. 6m).

In conclusion, we show that expression and activity of mitochondrial αKGDH, one of the rate-limiting enzymes of the TCA cycle, are reduced in chronically demyelinated axons. Reduced expression of αKGDH correlates with axonal damage and may be caused by hypoxic conditions, inflammation and oxidative stress.

## Discussion

This study provides a comprehensive overview of the expression and activity of key glycolytic, TCA cycle and lactate-metabolizing enzymes in MS lesions. We showed that expression levels of various metabolic enzymes were consistently upregulated in astrocytes and to a lesser extent in axons in active MS lesions. Astrocytes and axons in inactive MS lesions showed elevated expression of glycolytic enzymes as compared to NAWM. In inactive lesions, astrocytes express increased levels of lactate-producing enzymes, whereas the level of lactate-consuming enzymes was predominantly upregulated in axons. Finally, we observed that the expression of mitochondrial αKGDH was significantly decreased in demyelinated axons and correlated with axonal dysfunction.

Our immunohistochemical analyses revealed increased expression of key glucose - metabolizing enzymes (GME) in active MS lesions, suggesting that the activity of glycolytic and TCA cycle pathways is increased. In fact, we showed that αKGDH and LDH production capacities are increased in active MS lesions. In line with these observations, previous studies showed that astrocytes and axons in active MS lesions have an increased mitochondrial mass, enhanced OxPhos activity and express a wide variety of essential nutrient transporters [24, 29, 46]. Furthermore, imaging studies in MS patients indicate that both glucose and lactate metabolism is increased in active MS lesions [39, 41, 50]. Lactate levels were also found to be increased in the CSF and serum of MS patients and to correlate with disease progression [1, 35]. These studies suggest that the changes in GME expression levels we found are associated with alterations in the glycolytic and TCA cycle fluxes.

We previously demonstrated increased expression of nutrient transporters in reactive astrocytes in active MS lesions [29]. Here we found that GME expression showed a similar cellular distribution pattern as the nutrient transporters. Active MS lesions are characterized by abundant inflammation leading to enhanced cellular glucose metabolism whereas in vitro experiments demonstrated that exposure of astrocytes to pro-inflammatory cytokines increases glucose uptake and TCA cycle flux to a higher extent compared to neurons [4, 31]. Since protein synthesis takes place at the neuronal cell body and axons can reside at a great distance from the neuronal cell bodies, axonal structures cannot adapt protein expression as quickly as astrocytes, making them more dependent on the regulation of activity rather than transcription of proteins. This may explain why the most prominent changes in GME expression levels in active MS lesions were observed in astrocytes.

We observed marked astrocytic expression of enzymes involved in glycolysis in chronic lesions suggesting that astrocytes become more glycolytic in inactive MS lesions. High glycolytic rates coincide with enhanced production of lactate [45]. Here, we observed the same phenomenon in MS lesions since the LDHA/LDHB ratio in astrocytes was increased in inactive MS lesions as compared to surrounding NAWM. Thus, astrocytes seem to favour lactate production above utilization. Moreover, NAD^+^-dependent LDH activity was decreased in inactive MS lesions, which implies that the NADH-dependent LDH activity (*i.e.* production of lactate) is increased. These results are in line with previous studies which reported enhanced lactate levels in inactive MS lesions and increased astrocytic expression of the lactate transporter MCT1 [29, 40].

In inactive MS lesions, axons upregulated expression of glycolytic enzymes (HK2, PK) as well as TCA cycle enzymes (PDH and MDH) suggesting that axonal glucose metabolism is increased. In addition to glucose, axons can use lactate to fulfil their energetic needs [22]. Here, we showed that axons in inactive MS lesions have a lower LDHA/LDHB ratio as compared to NAWM, resulting in increased lactate utilization by demyelinated axons. Moreover, we found that demyelinated axons express abundant MCT2, despite an overall decrease in protein expression [29]. Under normal conditions, oligodendrocytes supply axons with lactate, however most oligodendrocytes are lost during the inflammatory attack in MS lesions [16, 22]. Our data suggests that astrocytes in inactive MS lesions may supply demyelinated axons with lactate. Future studies are needed to assess the functionality of astrocyte-axon coupling in vivo and should be directed to gain more insight in the role of lactate under pathological conditions.

Demyelinated axons are highly dependent on a sufficient supply of energy to maintain proper conduction, which may explain the upregulation of GME expression in demyelinated axons. Remarkably, we observed reduced αKGDH expression and NADH production capacity in axonal mitochondria in MS lesions. Double labelling of αKGDH with synaptophysin revealed that αKGDH expression is particularly reduced in axons with signs of impaired axonal transport. Interestingly, Mahad et al. previously observed decreased complex IV activity in a subset of dysfunctional axons [24]. Impaired αKGDH function can contribute to these changes in complex IV activity. As αKGDH is one of the rate-limiting enzymes of mitochondrial metabolism, reduced αKGDH activity can result in impaired mitochondrial energy metabolism and thereby contribute to neurodegeneration, which has been demonstrated in various studies [18, 21, 43]. Finally, we show that αKGDH activity and expression can be regulated upon exposure to a variety of stimuli [10, 27]. We found that, free radicals, TNF-α and IFN-γ, which are abundantly produced in active MS lesions, reduce αKGDH expression in vitro [3, 6, 14]. In inactive MS lesions, demyelinated axons are reported to be in a state of virtual hypoxia, which may also contribute to decreased αKGDH expression [42].

## Conclusions

In conclusion, our findings imply that in addition to reduced OxPhos activity, other key bioenergetic processes such as, glycolysis, TCA cycle and lactate metabolism are affected in MS lesions.

## Additional files

Additional file 1: Table S1. Antibodies. (DOCX 12 kb) Additional file 2: Figure S1. MS lesion classification. MS lesions are characterized by loss of myelin staining, here visualized by labelling of proteolipid protein (PLP) (A,C). In active lesions abundant MHCII positive immune cells occupy the lesion area (B). In chronic active lesions MHCII positive cells are mainly found around the rim of the lesion, but absent in the lesions center (D). (TIF 10733 kb) Additional file 3: Figure S2. The staining intensity of TCA cycle enzymes, PDH, αKGDH and MDH expression in inactive MS lesions compared to NAWM doesn’t correlate with the age of the patient (A) or MS subtype (B). (TIF 7705 kb) Additional file 4: Figure S3. Representative image of αKGDH production capacity in an active and inactive MS lesion with (A-C) and without appropriate substrate (B-D). Synaptophysin (E, red) and αKGDH (F,green) immunostaining in inactive MS lesions. Overlay is shown in G. (TIF 13088 kb)
