# Supplementary material for: Differential expression of glucose-metabolizing enzymes in multiple sclerosis lesions
Source: Acta Neuropathol Commun. 2015 Dec 4;3:79. doi: 10.1186/s40478-015-0261-8 (PMC4670517; doi:10.1186/s40478-015-0261-8)
Supplement: Additional file 1: Table S1. — Antibodies. (DOCX 12 kb) [file 40478_2015_261_MOESM1_ESM.docx]

Table S1. Antibodies

| **Antigen** | **Dilution** | **Antibody type** | **Source**^1^ |
| --- | --- | --- | --- |
| HK2 | 1:100 | Polyclonal | Sigma |
| PKM2 | 1:150 | Polyclonal | Novus Biological |
| PDH | 1:500 | Polyclonal | Abcam |
| OGDH | 1:150 | Polyclonal | Sigma |
| MCT2 | 1:1000 | Polyclonal | Sigma |
| MDH2 | 1:1000 | Polyclonal | Sigma |
| LDHA | 1:3000 | Polyclonal | Novus Biologicals |
| LDHB | 1:3000 | Polyclonal | Novus Biologicals |
| GFAP-Cy3 | 1:500 | IgG1 | Sigma |
| Pan-Neurofilament (SMI312) | 1:1000 | IgG1 | Covance |
| Porin | 1:500 | IgG2b | Abcam |

^1^Sources: Abcam, Camebridge, UK; Covance, Emeryville, CA; Novus Biologicals, Littleton, CO; Sigma-Aldrich, St Louis, MO
