# Supplementary figures and images for: Differential expression of glucose-metabolizing enzymes in multiple sclerosis lesions
Source: Acta Neuropathol Commun. 2015 Dec 4;3:79. doi: 10.1186/s40478-015-0261-8 (PMC4670517; doi:10.1186/s40478-015-0261-8)

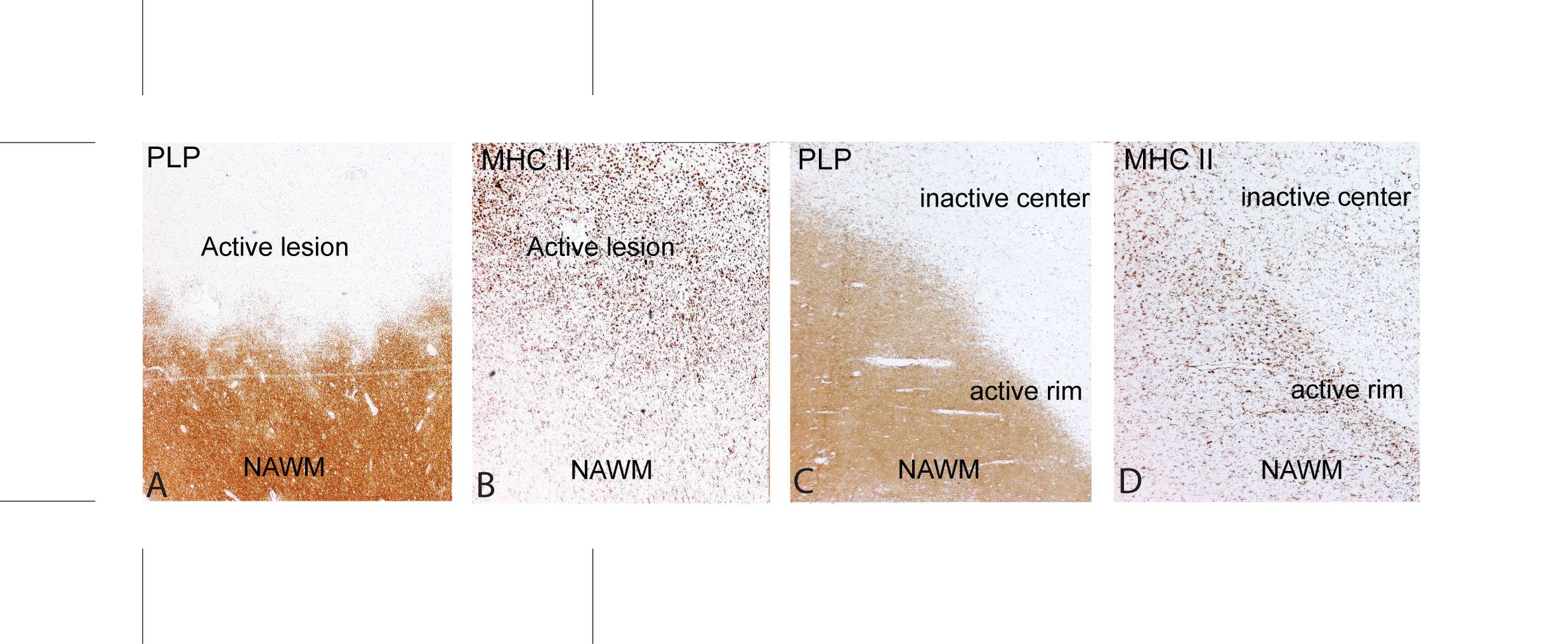

Supplement: Additional file 2: Figure S1. — MS lesion classification. MS lesions are characterized by loss of myelin staining, here visualized by labelling of proteolipid protein (PLP) (A,C). In active lesions abundant MHCII positive immune cells occupy the lesion area (B). In chronic active lesions MHCII positive cells are mainly found around the rim of the lesion, but absent in the lesions center (D). (TIF 10733 kb) [file 40478_2015_261_MOESM2_ESM.tif]

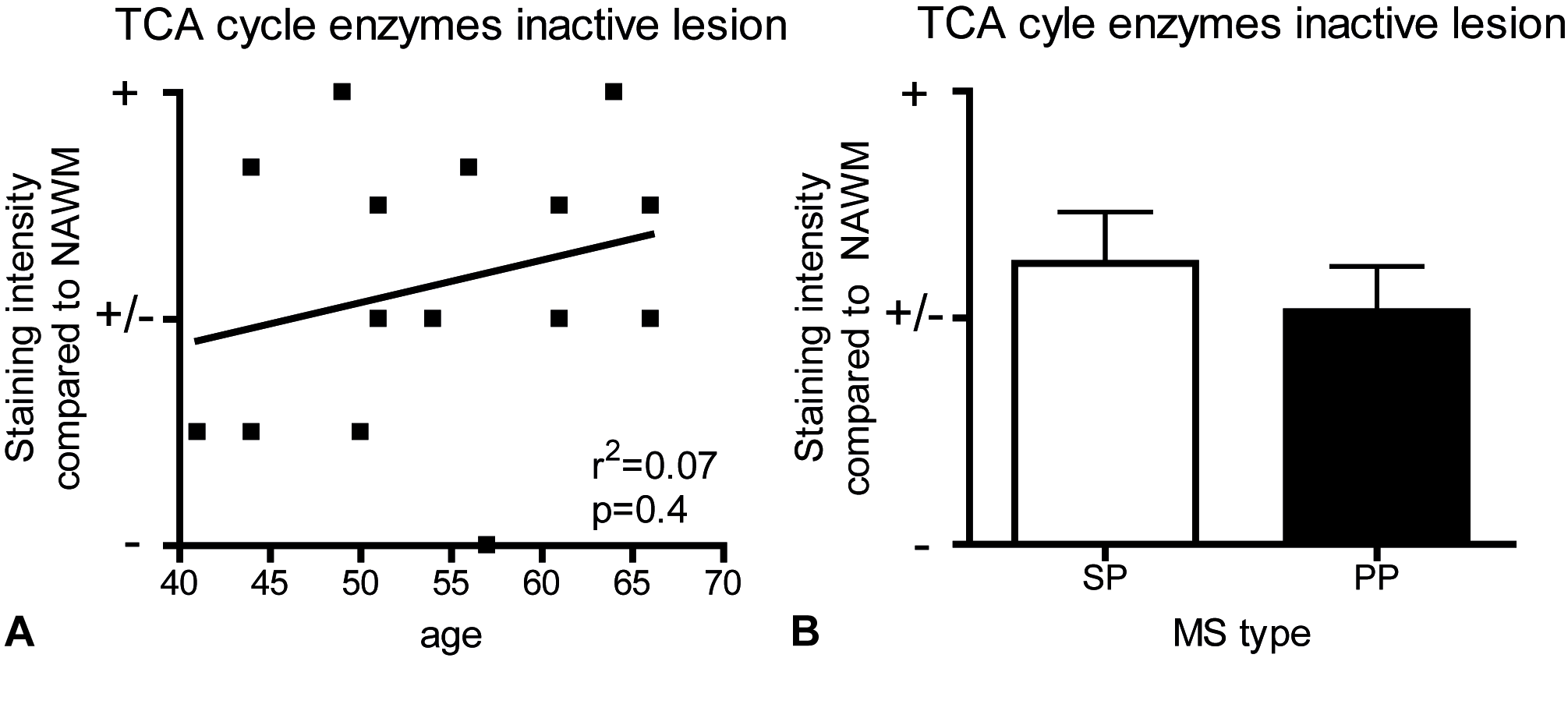

Supplement: Additional file 3: Figure S2. — The staining intensity of TCA cycle enzymes, PDH, αKGDH and MDH expression in inactive MS lesions compared to NAWM doesn’t correlate with the age of the patient (A) or MS subtype (B). (TIF 7705 kb) [file 40478_2015_261_MOESM3_ESM.tif]

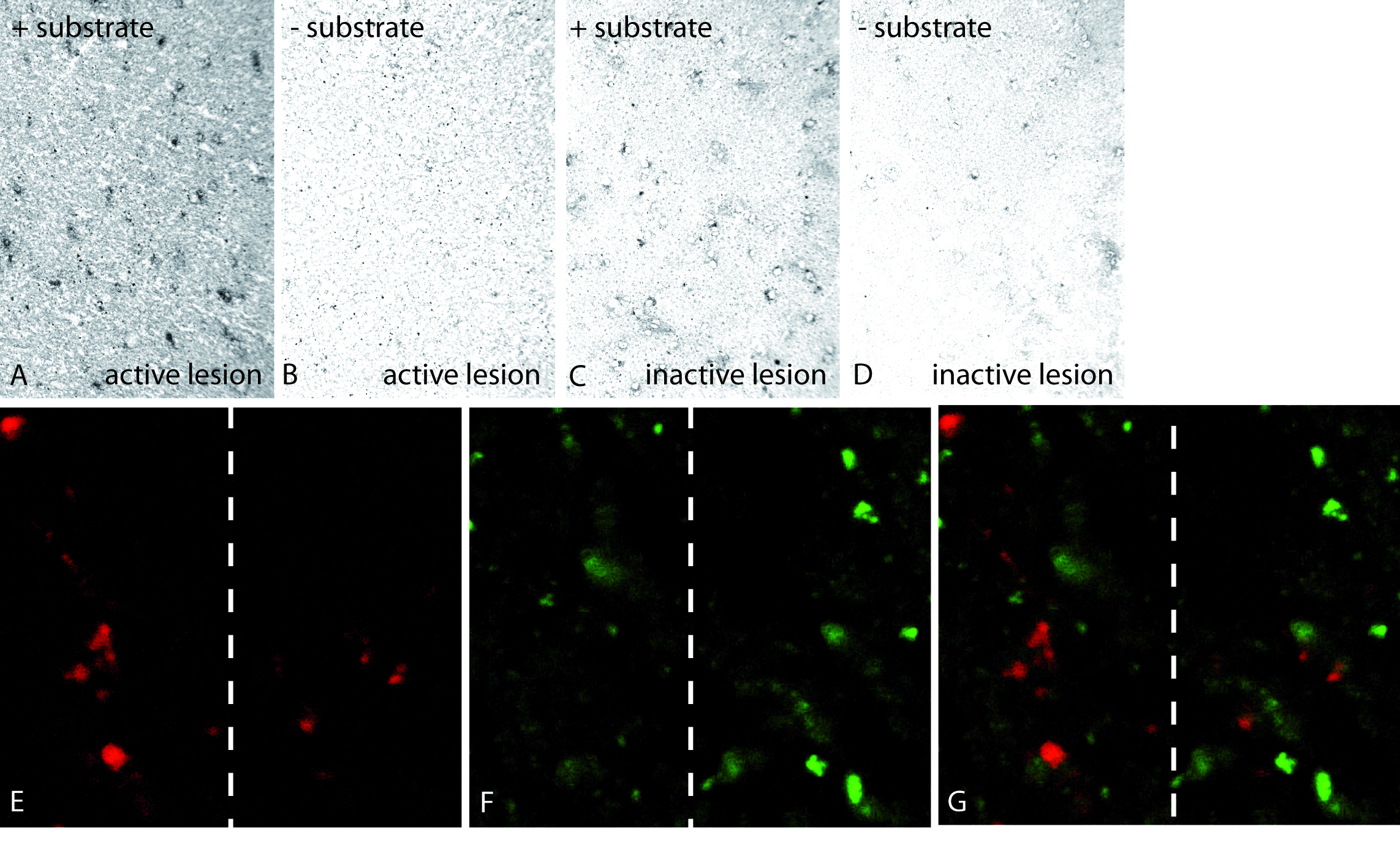

Supplement: Additional file 4: Figure S3. — Representative image of αKGDH production capacity in an active and inactive MS lesion with (A-C) and without appropriate substrate (B-D). Synaptophysin (E, red) and αKGDH (F,green) immunostaining in inactive MS lesions. Overlay is shown in G. (TIF 13088 kb) [file 40478_2015_261_MOESM4_ESM.tif]
